# Two previously proposed P_1_/P_2_-differentiating and nine novel polymorphisms at the *A4GALT *(*P*^*k*^) locus do not correlate with the presence of the P1 blood group antigen

**DOI:** 10.1186/1471-2156-6-49

**Published:** 2005-10-07

**Authors:** Åsa Hellberg, M Alan Chester, Martin L Olsson

**Affiliations:** 1 Division of Hematology and Transfusion Medicine, Department of Laboratory Medicine, Lund University and the Blood Centre, Lund University Hospital, Lund, Sweden

## Abstract

**Background:**

The molecular genetics of the P blood group system and the absence of P1 antigen in the p phenotype are still enigmatic. One theory proposes that the same gene encodes for both the P1 and P^k ^glycosyltransferases, but no polymorphisms in the coding region of the *P*^*k *^gene explain the P_1_/P_2 _phenotypes. We investigated the potential regulatory regions up- and downstream of the *A4GALT *(*P*^*k*^) gene exons.

**Results:**

P_1 _(n = 18) and P_2 _(n = 9) samples from donors of mainly Swedish descent were analysed by direct sequencing of PCR-amplified 5'- and 3'-fragments surrounding the *P*^*k *^coding region. Seventy-eight P_1 _and P_2 _samples were investigated with PCR using allele-specific primers (ASP) for two polymorphisms previously proposed as P_2_-related genetic markers (-551_-550insC, -160A>G). Haplotype analysis of single nucleotide polymorphisms was also performed with PCR-ASP. In ~1.5 kbp of the 3'-untranslated region one new insertion and four new substitutions compared to a GenBank sequence (AL049757) were found. In addition to the polymorphisms at positions -550 and -160, one insertion, two deletions and one substitution were found in ~1.0 kbp of the 5'-upstream region. All 20 P_2 _samples investigated with PCR-ASP were homozygous for -550insC. However, so were 18 of the 58 P_1 _samples investigated. Both the 20 P_2 _and the 18 P_1 _samples were also homozygous for -160G.

**Conclusion:**

The proposed P_2_-specific polymorphisms, -551_-550insC and -160G, found in P_2 _samples in a Japanese study were found here in homozygous form in both P_1 _and P_2 _donors. Since *P*^*2 *^is the null allele in the P blood group system it is difficult to envision how these mutations would cause the P_2 _phenotype. None of the novel polymorphisms reported in this study correlated with P_1_/P_2 _status and the P1/p mystery remains unsolved.

## Background

The P-related blood groups include four antigens that are predominantly of glycolipid nature and occur in related biosynthetic pathways [1].

**Figure 1:**
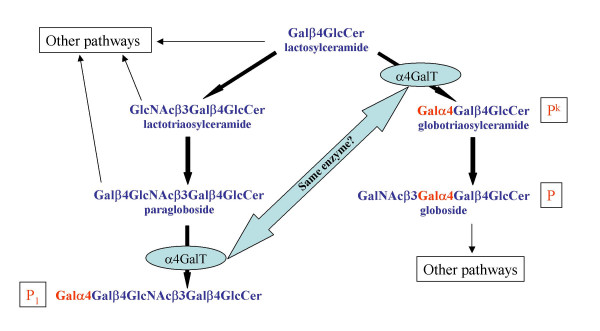
Biosynthetic pathways relating the P1, P and P^k^ glycolipids.

The GLOB blood group system [International Society of Blood Transfusion (ISBT) number 028] comprises the P antigen and the GLOB collection (ISBT number 209) includes the P^k ^antigen and also LKE that is not discussed further here [2]. The P1 antigen is assigned to ISBT system number 003. Five phenotypes depending on the presence or absence of the three antigens, P1, P and P^k^, are known (Table 1). The presence of all three antigens results in the P_1 _phenotype but absence of the P1 antigen causes the P_2 _phenotype. If both P1 and P are absent the phenotype P_2_^k ^arises. Absence of P but presence of P1 and P^k ^results in the P_1_^k ^phenotype. Absence of all three antigens results in the p phenotype. In each of the phenotypes naturally occurring-antibodies can arise against the missing antigen, invariantly so in the case of P and P^k ^but less frequently for P_1_. These phenotypes can be explained biochemically by the presence or absence of some of the enzymes shown to catalyse the pathways shown in Figure 1.

**Table 1:** The P/GLOB blood groups.

**Phenotype**	**Frequency**	**Antigen present on RBC**	**Antibodies in serum**
P_1_	20–90%^a^	P1, P^k^, P	none
P_2_	10–80%^a^	P^k^, P	Anti-P1
p	rare^b^	none	Anti-PP1P^k^
P_1_^k^	rare	P1, P^k^	Anti-P
P_2_^k^	rare	P^k^	Anti-PP1

^a ^These frequencies differ significantly between different populations. E.g. the frequency of P_1 _vs. P_2 _are virtually the opposite when Caucasians (80 vs. 20%) and Japanese (20 vs 80%) are compared.

^b ^While the frequency of this phenotype has been estimated at 1 per million, two population groups, Swedes and Amish people, have significantly higher numbers (e.g. 141 per million in Västerbotten county in Northern Sweden [21]).

The P1 antigen is present on hematopoietic cells [3,4] and other cells [5]. The strength of the antigen expression can differ from one person to another and it seems to be dependent on gene dosage [1]. Frequencies of the P_1 _phenotype vary in different ethnic groups, for example, ~80% of Caucasians are P_1_, compared to only ~20% of Asians [1]. This may be due to selective pressure since P1 and related antigens can act as cellular receptors for microorganisms and biotoxins [5].

Two genes that code for the P^k^- and P-synthesizing enzymes, 4-α-galactosyltransferase (α4GalT, Gb3 synthase) [6] and 3-β-*N*-acetylgalactosaminyltransferase (β3GalNAcT, globoside/Gb4 synthase) [7], respectively, have been cloned. Subsequently, numerous mutations have been found to explain the rare P_1_^k^, P_2_^k ^[8,9] and p [6,9-12] phenotypes.

The molecular genetic background of the P1 antigen remains unknown. Several theories exist, including one model suggesting that the same α4GalT is able to transfer galactosyl residues to both lactosylceramide and paragloboside but in order to use the latter as the acceptor, a regulatory protein is required [13]. Another model postulates the existence of two different enzymes, and thus two genes, requiring both of them to be inactivated to cause the p phenotype [13]. A third model proposes a single gene with three alleles, one allele coding for an α4GalT that can utilise lactosylceramide and paragloboside as acceptors, one allele using lactosylceramide only and the third allele coding for an inactive form of the transferase [14]. However, none of the known polymorphisms (109A>G, 903G>C, 987G>A) in the coding region of the *P*^k ^gene explains the P_1_/P_2 _phenotypes [6].

Recently, Iwamura *et al*. [15] suggested that transcriptional regulation caused by two polymorphisms (-551_-550insC, -160A>G) in the 5'-upstream region of the *P*^*k *^gene might be the reason for the P_1_/P_2 _phenotypes.

**Figure 2:**
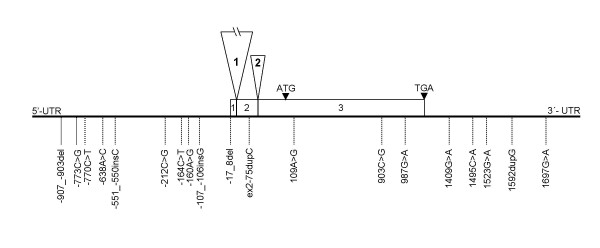
**Schematic picture of the exon/intron structure of the *A4GALT* (*P*^*k*^) gene and the positions of the 19 polymorphisms investigated**. The numbering systems used are as follows: The 5'-region and exon 1 are described using the same numbering as Iwamura *et al*. [15] for easy comparison. Intron 1 and exon 2 are numbered from the first nucleotide of each element, with the number being preceded by int1- or ex2-, respectively. The adenosine of the translation start codon ATG in exon 3 is defined as nt. 1. Nucleotides upstream from this (untranslated exon 3 and the 3'-region of intron 2) are given as negative numbers. The exons are represented by rectangles and the intervening introns by triangles. Exons 1–3 are 21, 145 and 1059 bp long, respectively and introns 1 and 2 are approximately 25 and 1.5 kbp long, respectively.

The *P*^*k *^gene was originally thought to comprise two exons, but recent GenBank depositions indicate the presence of three exons with the whole coding region in exon 3, as shown in Figure 2. Various publications have considered different transcription starting points resulting in different numbering of the same nucleotide positions. The numbers used here are described in the legend to Figure 2.

In this study we have investigated an extended sequence surrounding the coding region of the *P*^*k *^gene including untranslated exons and intronic portions as well as potentially regulatory regions 5' or 3'of the transcribed region. Contrary to a previous report [15], we found no clear-cut correlation with the P_1_/P_2 _phenotype, neither between previously described polymorphisms in the 5'-regulatory region, nor any of the novel polymorphisms reported in this study.

## Results

### Screening for the -551_-550insC and -160A>G polymorphisms by PCR-ASP

Seventy-eight samples were screened for the two genetic markers previously [15] suggested to cause the P_2 _phenotype. The results are summarized in Table 2. Two haplotypes, -550T;-160A and -551_-550insC;-160G, were found whilst the other theoretically possible haplotypes, 550T;-160G and -551_-550insC;-160A, were not detected in this study. Each of the 20 samples, that were phenotyped as P_2 _were homozygous for both -551_-550insC and -160A>G, which could indicate that these polymorphic positions were indeed P_2_-specific as proposed. However, 18 of the 58 P_1 _samples investigated were also homozygous for the same polymorphic markers. Of the remaining P_1 _samples, 32 were heterozygous at both nucleotide positions and only eight samples were homozygous for the proposed P_1_-specific combination, -550T;-160A. Twenty-nine of the 32 heterozygous samples were available for analysis by two haplotype-specific PCR reactions (-550T;-160A and -551_-550insC;-160G). All samples were positive in both PCR reactions, indicating that the samples were heterozygous for the combinations -550T;-160A and -551_-550insC;-160G and not for -550T;-160G and -551_-550insC;-160A. Additionally, 26 samples with the rare phenotypes P_1_^k ^(n = 3), P_2_^k ^(n = 3) and p (n = 20) were screened. One of the P_1_^k ^samples was homozygous for the genetic markers reported to be associated with the P_2 _phenotype, and the other two were heterozygous. Of the P_2_^k ^samples two had the expected polymorphisms (-551_-550insC;-160G), but the third P_2_^k ^was heterozygous and thus the first reported sample that lacks the P1 antigen in spite of a genotype not homozygous for the -551_-550insC and -160G markers. Sixteen of the 20 p samples were homozygous for -551_-550insC;-160G, thus consistent with their lack of P1, but one was heterozygous and three were homozygous for -550T;160A. Due to lack of available DNA from these rare individuals the haplotype-specific PCR was only run on one of the four heterozygous samples. As above, this sample was also positive in both PCR reactions.

**Table 2:** The occurrence of the -550T;-160A and -551_-550insC;-160G haplotypes in individuals with various P phenotypes.

		Combination of polymorphisms		
		
Phenotype	No.	-550T;-160A (homozygous)	-550T;-160A -551_550insC;-160G (heterozygous)	-551_-550insC;-160G (homozygous)
P_1_	58	8	32	18
P_2_	20	0	0	20
P_1_^k^	3	0	2	1
P_2_^k^	3	0	1	2
p	20	3	1	16

### Amplification of the 5'-, 3'- and coding regions of the *P^k ^*gene for DNA sequencing

Ten of the 18 P_1 _samples with P_2_-associated polymorphisms (-551_-550insC;-160G) were chosen for further investigation by sequencing the 5'-region and the 3'-region, as were all eight P_1_(-550T;-160A) and nine of the 20 P_2 _samples. In three samples from each category the whole *P*^*k *^gene reading frame, located in exon 3, was also sequenced.

In the 5'-region upstream of exon 1, four novel polymorphisms were detected compared to a sequence deposited in GenBank (accession number AL049757). These findings comprised a substitution (-770C>T), an insertion (-107_-106insG) and two deletions (-907_-903del and -17_8del). Interestingly, the latter deletion is located across the border of the 5'-region and exon 1.

In the 3'-UTR, five new polymorphisms were found, four of which were substitutions (1409G>A, 1495C>A, 1523G>A, 1697G>A) and one was an insertion (1592dupG). Figure 2 shows the relative positions of the polymorphisms investigated.

**Figure 3:**
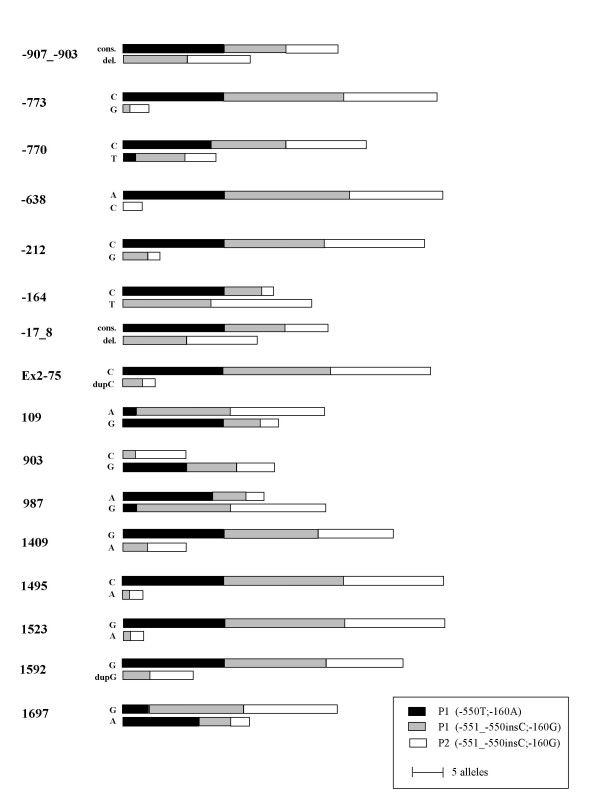
**The distribution of the polymorphic variants in the three different sample categories: P1(-550T;-160A), P1(-551_-550insC;-160G) and P2**. The diagram shows data of 16 polymorphisms investigated. The data for the 5'- and the 3'-regions were obtained by sequencing while most of the data for exon 3 originated from PCR-ASP analysis. Eight, P1(-550T;-160A), ten P1(-551_-550insC;-160G) and nine P2 samples were investigated. However, for the 903C>G polymorphism the corresponding numbers were five, five and seven samples. In position -107_-106 all investigated samples had three guanosines instead of two as reported in the reference sequence (AL049757). We therefore do not consider this position as polymorphic in our analysis and it is consequently not included in this figure. cons. = consensus.

The distribution of the polymorphisms in the three different categories, P1(-550T;-160A), P1(-551_-550insC;-160G) and P2 are shown in Figure 3. As can be seen, none of the polymorphisms is a genetic marker specific for the P_2 _phenotype. On the other hand, individuals from the P1(-550T;-160A) category are homozygous for 12 of the 16 investigated polymorphisms. However, when individuals from the P1(-551_-550insC;-160G) samples are included such pattern is no longer evident. It can also be noted that the frequency of some of the variants appears to be relatively low, less than ~10 %, for six of the polymorphic sites analyzed.

While sequencing exon 3, an unexpected mutation, 441G>A, was encountered in a P1 sample of African origin. No other samples, including two of African descent, examined in this study had this particular mutation, which would not alter the amino acid sequence, or any other new polymorphisms.

### Haplotype analysis of SNPs in the reading frame and a polymorphism in the 3'-UTR using PCR-ASP

We also performed PCR-ASP utilising polymorphisms in exon 3 and in the 3'-UTR of the *P*^*k *^gene to determine the *cis/trans *linkage of SNPs and to establish if combinations of polymorphisms correlated with specific alleles. The SNPs chosen were two of those previously described, i.e. 109A>G and 987G>A. Analysis of the polymorphism 903C>G was considered unnecessary due to its proximity to nt. 987. In addition, of the new polymorphisms the one furthest downstream (1697G>A) was analyzed. The results are summarized in Table 3. None of the haplotypes correlated well with the P_1_/P_2 _phenotypes although 83% of the alleles among the P2 samples and 75% of the P1(-551_-550insC;-160G) category samples had the A-1 haplotype, whilst in the P1(-550T;-160A) category samples only 12.5% had this partial haplotype (B-1). The remaining alleles in this latter category consisted of 12.5% of the rare B-3 and 75% of the B-2 haplotype.

**Table 3:** Correlation of phenotype and haplotype

Phenotype	No. of samples	Polymorphism					Haplotype	No. of haplotypes
				
		-550	-160	109	987	1697		
P_1_	10	insC	G	A	G	G	A-1	15
		insC	G	G	A	A	A-2	5
								
P_1_	8	T	A	A	G	G	B-1	2
		T	A	G	A	A	B-2	12
		T	A	G	A	G	B-3	2
								
P_2_	9	insC	G	A	G	G	A-1	15
		insC	G	G	A	A	A-2	3

## Discussion

The molecular background of the P blood group system has been a subject of speculation since its discovery. Antigens formed as a result of closely related biosynthetic pathways are now known to arise from independent genetic loci. However, the current paradox revolves around the 4-α-galactosyltransferase (α4GalT) that synthesises the P^k ^antigen and possibly also the P1 antigen. If it is indeed exactly the same enzyme synthesizing both, then the P_2 _and P_2_^k ^phenotypes should not exist, as the P1-synthesizing α4GalT that is lacking in these individuals should also result in the loss of the P^k ^and, subsequently, P antigens. The absence of the P1 antigen is not due to a defect in the biosynthesis of paragloboside since this glycolipid is a major precursor of the erythrocyte ABH antigens that are unaffected by the P_1_/P_2 _status. The absence of P1 antigen in the P_2 _phenotype is likely to be caused by inefficient or absent glycosyltransferase activity that can be due to a structural defect in the enzyme itself or more indirectly due to various other factors necessary to ensure the enzyme's optimal expression, localisation and efficiency[1]. Studies are currently in progress to analyse *A4GALT *mRNA by real-time PCR and other methods.

The P1 and P^k ^antigens both share the same terminal carbohydrate structure and therefore the question arises whether the same enzyme is, or is not, synthesizing both antigens. Various theories exist but so far none has been proven correct. Recently Iwamura *et al *[15] proposed that the P1 synthase is identical to the P^k ^synthase and that the phenotypic difference may be caused by two polymorphisms, -160A>G and -551_-550insC, found in the 5'-upstream regulatory region. Unfortunately, these authors were not able to show any functional effects of these two polymorphisms, at least not when tested in a fibroblast cell line. As they commented themselves, a haematopoietic cell line, preferentially even an erythropoietic one, would have been the optimal choice for the challenge but was technically difficult. Iwamura *et al*. also demonstrated the presence of cryptic and intracellular P1 antigen in cells from individuals known to have the P_2 _red blood cell phenotype. Whether this surprising finding is method-dependent or may differ between populations is currently unclear.

Our data indicate that an individual's P1/P2 status is not due to the -551_-550insC;-160G haplotype, contrary to the previously published indication [15]. However, it is striking that all (except one P_2_^k^) samples with the P_2 _phenotype tested here have the same -551_-550insC;-160G haplotype as all ten P_2 _samples tested by Iwamura *et al*. This clearly argues that the genetic linkage between the *A4GALT *locus and P2 status must be relatively strong. The results shown in Figure 3 indicate that none of the 16 polymorphic markers investigated can predict the serologically determined P1/P2 status. It is also interesting to note the high frequency with which SNPs occur in the *A4GALT *gene, as opposed to the gene coding for the next glycosyltransferase in this biosynthetic pathway, the P synthase, in which we have found no variations at all. The only exceptions are rare mutations causing the P_1_^k ^and P_2_^k ^phenotypes [8,9]. Despite the apparent variability in the *A4GALT *gene, the current study suggests that a limited number of haplotypes, with the A-1 and B-2 haplotypes being the predominant ones in the Swedish population, may constitute genetic clusters within which further variation has arisen (as judged by the lack of homogeneity within each group, see Table 3), still without obvious correlation to the blood group phenotype.

However, all this does not differentiate between the one-gene theory and the possibility of a tightly coupled independent locus responsible for P1 antigen expression. Since there are no apparent α4GalT homologues in this genetic region this may imply that a hypothetical second closely linked gene would give rise to *either *a regulatory molecule modifying the acceptor specificity of the P^k^-synthesizing α4GalT (analogous to lactose synthetase [16]) *or *a chaperone type of molecule to make a fraction of the α4GalT molecules more suitably located/positioned for P1 synthesis. The finding of intracellular P1 antigen in P_2 _individuals [15], would tend to support the latter possibility. Chaperones have been shown to be involved in processes related to glycosyltransferase action [17] but it is somewhat difficult (although not impossible) to imagine an α4GalT-specific chaperone to be the solution for this long-standing enigma.

## Conclusion

The study of potential regulatory regions surrounding the *P*^*k *^coding sequence revealed nine previously unreported polymorphisms but none of them correlated with the P_1_/P_2 _red blood cell phenotypes. Two polymorphisms, -551_-550insC and-160A>G, suggested to cause the P_2 _phenotype in Japanese individuals [15] were found in homozygous form also in P_1 _samples in this study and since the P_2 _is the null phenotype of this blood group system, it is therefore very unlikely that these mutations cause the P_2 _phenotype.

## Methods

### Blood samples and DNA preparation

Samples with the P_1 _(n = 58) and P_2 _(n = 20) phenotypes were chosen from our in-house panel of test erythrocytes. The majority of the donors are of Swedish origin but a few are of Asian or African descent. Three P_1_^k^, three P_2_^k ^and 20 p samples, genetically characterized in our laboratory, were also included for screening purposes [8,9,12,18]. The erythrocyte phenotype was determined by standard serological techniques.

DNA was prepared from EDTA blood using a simple salting-out method for small volumes modified from Miller *et al*. [19], or Qiagen QIAmp Blood Extraction kit (Qiagen GmbH, Hilden, Germany). The DNA was dissolved in H_2_O at a concentration of 100 ng/μl.

### Screening for the -551_-550insC and -160A>G polymorphisms by PCR-ASP

**Figure 4:**
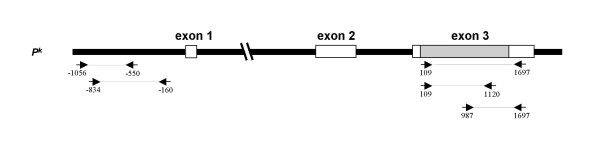
**Schematic representation of the primers used to identify the polymorphisms at -551_-550insC and -160A>T, described to cause the P_2 _phenotype. **The figure also shows combinations and positions for the primers used to detect a linkage phase of SNPs in the reading frame with a polymorphism in the 3'-UTR. For size reference, see Figure 2.

All oligonucleotide primers used in the study were synthesized by DNA Technology ApS (Aarhus, Denmark) and the sequences are shown in Table 4. PCR with allele-specific primers designed to detect the polymorphisms at -551_-550insC and -160A>T, described to cause the P_2 _phenotype [15] was performed. For all heterozygous samples double allele-specific amplification, -551_-550insC;-160G and -550T;160A, were performed. The primer combinations used are listed in Table 5 and the locations in Figure 4. Primers were mixed with 100 ng of genomic DNA, 2 nmol of each dNTP, 2% glycerol, 1% cresol red and 0.5 U of AmpliTaq Gold (Perkin Elmer/Roche Molecular Systems, Branchburg, NJ, USA) in 10 × PCR buffer with 15 mM MgCl_2_. The final reaction volume was 11 μl. Thermocycling was undertaken in GeneAmp PCR system 2400/2700 (Perkin-Elmer/Cetus, Norwalk, CT, USA) under PCR conditions described in Table 5.

**Table 4:** Oligonucleotide primers used in this study.

Primer name	Nucleotide sequence (5'→3')	Function
Pk-5'-(-1056)-F^†^	ACAGCCTGTGATGGGAATGAC	a, b, e
Pk-5'-(-740)-R^†^	TTGAGTGCTGACGCCCATCC	b
Pk-5'-(-834)-F	TGGGCACCCATTGAGTGCCA	b, e
Pk-5'-(-550)-R	ACCTCGCCCCATCTTCACAC	e
Pk-5'-(550T)-F	GAACAAATTACCAATAGCAATATGT	e
Pk-5'-(-550insC)-R	CCTCGCCCCATCTTCACAGC	e
Pk-5'-(550insC)-F	AACAAATTACCAATAGCAATATGCT	e
Pk-5'-(-477)-F	GCGGCGTTAAGGATACAGCAA	b
Pk-5'-(-418)-R	CTGATCCCACCGCCTCCTG	b
Pk-5'-(-235)-F	GCGCTCCCTACCTGTTGGC	b
Pk-5'-(-131)-F	GGACCGGGACCCGCAGGG	a, b
Pk-5'-(-160Gmis)-R	CCCGGTCCCCAGAGCACC	e
Pk-5'-(-160Amis)-R	CCCGGTCCCCAGAGCACT	e
Pk-5'-(-160G)-R	CCGGTCCCCAGAGCCCTC	e
Pk-5'-(-160A)-R	CCCGGTCCCCAGAGCCCTT	e
Pk-int1-35-R	CGTCCCCCGCAACATCGGC	b
Pk-int1-160-R	GCACAAATGTCGCCTCCAGAA	a, b
Pk-ex2-74_75insC-F	AGGTCGGCTGCTGAGCCCA	e
Pk-int2-R	GGGTGCAACCTGATTGCTAAG	e
Pk-109G-F	TTCACGTTTTTCGTCTCCATCG	e
Pk-109A-F	TTCACGTTTTTCGTCTCCATCA	e
Pk-987G-F	CACGCGGTTCGAGGCCACG	e
Pk-987A-F	CACGCGGTTCGAGGCCACA	e
Pk-1120-R	GGAAGGGCGGCCCAGTGC	e
Pk-1006-F	CCAGGGCACTGCTGGCCC	c, d
Pk-1253-F	GGACAGTGTCCTGTCTCGAG	d
Pk-1697G-R	CCTGTCTGAGGGAAGGGGC	e
Pk-1697A-R	CCCTGTCTGAGGGAAGGGGT	e
Pk-1791-R	TTATTCTATTGATTATTCTCCTGTG	d
Pk-1881-R	CCCCGTCAGAAGAATGGAGC	c, d
HGH-F	TGCCTTCCCAACCATTCCCTTA	f
HGH-R	CCACTCACGGATTTCTGTTGTGTTTC	f
JK-781-L-F	GCATGCTGCCATAGGATCATTGC	f
JK-943-L-R	GAGCCAGGAGGTGGGTTTGCC	f
MO-21	GGTGAGAGAAGGAGGGTGAG	f
MO-31	CCAGCACCCCGGCCAGCA	f

^† ^F = Forward. R = Reverse. The underlined nucleotide is mismatched for better specificity.

a. used for amplification of the 5' regulatory region.

b. used for sequencing of the 5' regulatory region.

c. used for amplification of the 3'-UTR.

d. used for sequencing the 3'-UTR.

e. used for PCR-ASP.

f. used as internal control.

**Table 5:** Oligonucleotide primer combinations and PCR conditions (previously not described) used for PCR-ASP in this study.

Primer combination	Specific primer (pmol)	Control primer			DMSO (%)	Annealing temperature (°C)	Extension time (sec)
					
		HGH-F/R (pmol)	JK-L-F/R (pmol)	MO-21/31 (pmol)			
Pk-5'-(-1056)-F	5	1				64	40
Pk-5'-(-550)-R	5						
							
Pk-5'-(-1056)F	5	1				64	40
Pk-5'-(-550insC)-R	5						
							
Pk-5'-(-834)-F	7.5		0.5		4	62	60
Pk-5'-(-160A-mis)-R	7.5						
							
Pk-5'-(-834)-F	5		0.5		3	62	60
Pk-5'-(-160G-mis)-R	5						
							
Pk-5'(-550insC)-F	10			0.75		64	40
Pk-5'-(-160G)-R	10			0.75			
							
Pk-5'(-550T)-F	7.5			0.5		63	40
Pk-5'-(-160A)-R	7.5			0.5			
							
Pk-109A-F or Pk-109G-F	6	0.4			2	65	90
Pk-1697A-R or Pk-1697G-R*	6						
							
Pk-987A-F	5	0.4				66	60
Pk-1697A-R	5						
							
Pk-987G-F	5	0.4				66	60
Pk-1697G-R	5						
							
Pk-109A-F or Pk-109G-F	7.5		0.5			64	60
Pk-1120-R**							

Denaturation was carried out at 96°C for 7 min followed by 35 cycles at 94°C for 30 s. The annealing time was 30 s.

* Four double allele-specific amplifications were performed.

** These combinations were used for allele-specific amplifications for sequencing purposes.

### Amplification of the 5'-, 3'- and coding regions of the *P^k ^*gene for DNA sequencing

The coding region, exon 3, in the *P*^*k *^gene was amplified with the primer pair Pk-(-140)-F and Pk-1120-R in the Expand High Fidelity PCR System (Roche Molecular Systems, Pleasanton, CA, USA) and sequenced as previously described [12].

The 5'-regulatory region of the *P*^*k *^gene was amplified with primers Pk-5'-(-1056)-F and Pk-int1-160-R and Pk-5'-(-131)-F and Pk-int1-160-R. Amplification was performed in a reaction volume of 22 μL with four pmol of each primer, 2 nmol of each dNTP, 100 ng of genomic DNA, GC-rich enzyme mix (0.5 U per reaction), GC-rich resolution solution and buffer with a final MgCl_2 _concentration of 1.5 mM (GC-rich PCR System, Roche Diagnostics GmbH, Mannheim, Germany). Thermocycling was undertaken in GeneAmp PCR system 2400/2700 (Perkin-Elmer/Cetus): Initial denaturation at 96°C for 7 min followed by 10 cycles at 94°C for 30 s, 62°C for 30 s and 72°C for 1 min and then 25 cycles at 94°C for 30 s, 60°C for 30 s and 72°C for 1 min.

For the 3'-region of the *P*^*k *^gene 5 pmol of primers Pk-(1006)-F and Pk-(1881)-R were mixed with 100 ng of genomic DNA, 2 nmol of each dNTP, 2% glycerol, 1% cresol red and 0.5 U of AmpliTaq Gold (Perkin Elmer/Roche Molecular Systems) in 10 × PCR buffer with 15 mM MgCl_2_. The final reaction volume was 11 μl. PCR was run at 96°C for 7 min followed by 35 cycles at 94°C for 30 s, 64°C for 30 s and 72°C for 1 min.

PCR products were excised from 3% agarose gels (Seakem, FMC Bioproducts, Rockland, ME, USA) stained with ethidium bromide (0.56 mg/l gel, Sigma Chemicals, St. Louis, MO, USA) following high-voltage electrophoresis and purified using Qiaquick gel extraction kit (Qiagen). The Big Dye Terminator Cycle Sequencing kit (Applied Biosystems, Foster City, CA, USA) and an ABI PRISM 310 Genetic Analyser (Applied Biosystems) were used for direct DNA sequencing with capillary electrophoresis and automated fluorescence-based detection according to the manufacturer's instructions. Besides the PCR primers, internal primers were used as sequencing primers, see Table 4. To avoid detection of artefacts, sequencing was performed on both strands and using independently obtained fragments.

### Detection of an insertion in exon 2 and linkage of SNPs in the reading frame with a polymorphism in the 3'-UTR using PCR-ASP

PCR-ASP for a previously found insertion, 75dupC in exon 2 (then believed to be exon 1), was also performed as described [12]. PCR-ASP was performed to investigate if the polymorphisms at nt. 109, 987 and 1697 were present in an allele-specific pattern. The reaction mixtures comprised 100 ng of genomic DNA, 2 nmol of each dNTP, 2% glycerol, 1% cresol red and 0.5 U of AmpliTaq Gold (Perkin Elmer/Roche Molecular Systems) in 10 × PCR buffer with 15 mM MgCl_2_. The final reaction volume was 11 μl. Primer combinations and PCR conditions are described in Figure 4 and Table 5.

## List of abbreviations

ASP, allele specific primer

bp, base pairs

ins, insertion

del, deletion

kb, kilo bases

PCR, polymerase chain reaction

SNP, single nucleotide polymorphism

UTR, untranslated region

ISBT, International Society of Blood Transfusion.

## Authors' contributions

ÅH carried out the experimental studies and participated in the discussion and preparation of the manuscript. AC participated in the discussion and preparation of the manuscript. MLO contributed to the design and coordination of the study and participated in preparation of the manuscript. All authors read and approved the final manuscript.
